# Neuroangiostrongyliasis Infection Risk Near Preschool Centres in Mallorca, Spain: A Pilot Micro‐Epidemiological Study

**DOI:** 10.1111/zph.13228

**Published:** 2025-06-02

**Authors:** Sebastià Jaume‐Ramis, Phoebe Rivory, Irene Serra Velázquez, Jan Šlapeta, Claudia Paredes‐Esquivel

**Affiliations:** ^1^ Mediterranean Parasitology and Ecoepidemiology Research Group (PaEMed), Biology Department University of the Balearic Islands Palma Spain; ^2^ Sydney School of Veterinary Science, Faculty of Science The University of Sydney Camperdown New South Wales Australia; ^3^ Sydney Infectious Diseases Institute The University of Sydney Camperdown New South Wales Australia; ^4^ CIBER Enfermedades Infecciosas CIBERINFEC‐MICINN‐ISCIII Madrid Spain

**Keywords:** *Angiostrongylus cantonensis*, children, meningitis, paediatric, rat lungworm, schools

## Abstract

**Introduction:**

Neuroangiostrongyliasis, caused by *Angiostrongylus cantonensis* , is a globally emerging zoonosis, with Spain being the only endemic country in Europe. Human infection occurs through ingestion of gastropods or paratenic hosts carrying third‐stage larvae, often leading to eosinophilic meningoencephalitis. Alternative routes such as the ingestion of gastropod mucus or contaminated water have been proposed as potential routes of infection. Young children, particularly those under 5 years old, are at higher risk of neurological complications. This study aimed to assess the risk of neuroangiostrongyliasis transmission in preschool children at an endemic site in Mallorca through a micro‐epidemiological approach.

**Methods:**

Gastropods from an area where an infected rat was detected were identified and screened for *A. cantonensis* , with parasitic loads quantified via qPCR. Positive samples were digested to confirm the presence of L3. The distribution of infected gastropods was mapped and analysed for spatial clustering. Teacher surveys were conducted to assess exposure risks within the school.

**Results:**

Overall prevalence in gastropods was 7.38%, with only slugs testing positive, showing a higher prevalence (28.2%). *Milax nigricans*, *Deroceras reticulatum* and *D. panormitanum* were confirmed as intermediate hosts, with *D*. *panormitanum* representing a new global host record. L3 larvae were observed in the three slug species. Larval loads ranged from 1 to 20,000 L3s. Infected slugs exhibited a clustered distribution near the positive rat location. Despite the limited epidemiological surveillance, teacher surveys revealed that children place gastropods in their mouths during outdoor activities.

**Conclusions:**

Multiple risk factors for neuroangiostrongyliasis were identified in an endemic area of Mallorca. Enhanced surveillance, improved diagnostics, treatment protocols and public health interventions are needed to prevent paediatric infections in Spain.

**Summary**

- The study site presents multiple risk factors for neuroangiostrongyliasis in schoolchildren, including the circulation of infected rats, proximity to slugs carrying high parasitic loads, reports of children placing gastropods in their mouths and staff unawareness.
- Further surveillance is needed in other endemic regions of Spain—the only endemic country in Europe—to protect children under 5, who are at greater risk of severe infections and neurological complications.
- We recommend that policymakers increase public awareness and establish prevention strategies to reduce transmission risks in emerging endemic regions.

## 1 Introduction

The rat lungworm disease, an emerging zoonosis caused by the neurotropic nematode *Angiostrongylus cantonensis* , was first identified in the Indo‐Pacific region. Since then, its geographic spread has accelerated, with reports on every continent except Antarctica (Cowie et al. 2022). Europe was long considered non‐endemic until 2019, when the parasite was detected in Tenerife (Foronda et al. 2010), an overseas territory of Spain. Subsequent discoveries in Mallorca (Paredes‐Esquivel et al. 2019) and Valencia, mainland Spain (Galán‐Puchades et al. 2022) suggest an ongoing expansion across the continent (Paredes‐Esquivel et al. 2023).

The life cycle of *Angiostrongylus cantonensis* involves two primary hosts: rats and gastropods as definitive and intermediate hosts, respectively. Rats become infected by ingesting gastropods containing the third‐stage larvae (L3), which then migrate to the central nervous system (CNS) before reaching the pulmonary arteries. Transmission is further facilitated by a wide range of paratenic hosts (Turck et al. 2022). Birds and mammals, including humans, serve as dead‐end hosts, where the larvae exhibit neurotropism, but fail to complete their migration. This triggers a strong inflammatory response, often leading to eosinophilic meningitis (neuroangiostrongyliasis, NA) and other CNS complications (McAuliffe et al. 2019). In some cases, larvae may undergo aberrant migration, resulting in ocular angiostrongyliasis (Dumidae et al. 2023).

To date, at least 2800 cases of human angiostrongyliasis have been officially reported; however, the true number is likely higher, with estimates closer to 7000 cases (Cowie et al. 2022). In adults, most cases resolve spontaneously or with supportive treatment and fatalities are rare. However, severe non‐specific cases occur more frequently in children, facing an increased risk of severe disability and higher mortality rates compared to adults (Evans‐Gilbert et al. 2014; Hwang and Chen 1991; McAuliffe et al. 2019; Morton et al. 2013).

Most NA outbreaks have been linked to the consumption of raw gastropods or paratenic hosts, such as prawns, either as part of traditional diets or due to cultural and religious practices (Cowie et al. 2022). Nevertheless, infections also occur in regions where raw gastropod consumption is uncommon (Evans‐Gilbert et al. 2014; Johnston et al. 2019; Morton et al. 2013). The ability of larvae to emerge from intermediate hosts either through gastropod mucus or from drowned gastropods has been demonstrated in several studies (Howe et al. 2019; Modrý et al. 2021; Rollins et al. 2023; Šipková et al. 2024). These findings suggest the possibility of alternative infection routes, including contaminated water, food, or contact with contaminated hands. Paediatric cases are typically associated with the accidental or inadvertent ingestion of intermediate hosts (Hasan et al. 2025) or, surprisingly, with ingestion as part of a dare or bet (Cowie et al. 2022).

This study aims to understand the transmission risk of angiostrongyliasis in an endemic area of Mallorca at a micro‐epidemiological level, with a focus on its proximity to preschool‐aged children. The investigation is done under the current context of the widespread circulation of *A. cantonensis* across at least 14 localities from Mallorca and the absence of established preventive measures in the country. To date, no autochthonous human cases have been reported in Europe so far.

## 2 Methods

### 2.1 Risk Map Design and Gastropod Sampling

The study area, covering a 1 km radius, was defined around the site where two infected rats were previously detected during ongoing *A. cantonensis* monitoring on Mallorca, as well as due to its proximity to preschool‐aged children. Following the similar approach as described by Rivory et al. (2023), the area was divided into 200 × 200 m grids, each assigned with Cartesian coordinates (*x*, *y*).

Gastropod sampling took place in January 2024, consistent with previous observations of *A. cantonensis* seasonality during the autumn and winter months (Delgado‐Serra et al. 2024). When possible, up to four snails/slugs were manually collected from each complete grid (fully formed squared grids) and stored individually in labelled zip‐lock bags to prevent cross‐contamination. The collected gastropods were subsequently stored at −20°C for further analysis.

### 2.2 Gastropod Identification

The identification of snails and slugs was primarily based on morphological characteristics (Beckmann 2007; Cadevall and Orozco 2016). When this proved inconclusive, a molecular‐based approach was used. Genomic DNA was isolated from foot tissue snips as described in Jaume‐Ramis et al. (2023). The cytochrome c oxidase I (COI) barcode region was then amplified and sequenced using the primers developed by Folmer et al. (1994).

PCR was performed using a Veriti Thermal Cycler (Applied Biosystems, USA) under the following conditions: an initial denaturation step at 95°C for 3 min, followed by 35 cycles at 95°C for 30 s, 50°C for 30 s and 72°C for 1 min, with a final extension step at 72°C for 10 min. Each 25 μL contained 1 μL of each primer at 10 μM, 1 μL of the template DNA, 12.5 μL of the Supreme NZYtaq II 2× Green Master Mix (Nzytech, Portugal), 8.5 μL of MilliQ water and 1 μL of MgCl_2_ 50 mM. Amplified products were purified using the NZY Gelpure kit (Nzytech, Portugal) and sent to Macrogen (Spain) for Sanger sequencing using the forward PCR primer only. The resulting sequences were cleaned and subjected to BLAST analysis (https://blast.ncbi.nlm.nih.gov) for species identification.

### 2.3 Detection and Quantification of *A. cantonensis* in Gastropod Tissue Using AcanR3990 qPCR

To detect the presence of *Angiostrongylus* larvae, a probe‐based quantitative qPCR assay targeting a repeat sequence on contig 3990 (AcanR3990) of *Angiostrongylus* was used (Sears et al. 2021) on the tissue snip DNA samples obtained from all gastropods. The analysis was performed at the University of Sydney, Laboratory of Veterinary Parasitology. Reactions were run at a final volume of 10 μL, including 2 μL of template and 5 μL Luna Universal Probe qPCR Master Mix (New England Biolabs, Australia). Forward and reverse primers were added at a final concentration of 0.4 μM and the probe at a final concentration of 0.1 μM (Integrated DNA Technologies Inc., Australia). All qPCR runs included an extraction control and a PCR‐grade water no‐template control. To estimate the load of L3, each run was also accompanied by five standards, which comprised 10‐fold dilutions of DNA from *A. cantonensis* L3s equivalent to 10, 1, 0.1, 0.01 and 0.001 × L3/s. Reactions were performed in a CFX Opus Real‐Time PCR Systems (Bio‐Rad Laboratories Inc.) with the following cycling conditions: 95°C for 3 min; and 40 cycles of 95°C for 5 s and 60°C for 15 s. Amplification curves and Cycle Threshold (*C*_T_)‐values were recorded using CFX Maestro Software 2.3 (Bio‐Rad Laboratories Inc.) and adjusted manually to improve the consistency of standard curves. Samples with *C*_T_ values lower than the 0.001 × L3 standard in their respective runs were considered positive; all other samples were considered negative.

### 2.4 Data and Statistical Analyses

Data were recorded in Microsoft Excel 2021 and qPCR results were interpreted with descriptive analyses. Confidence limits for sample proportion (Wald's method) were used to determine the 95% confidence interval (CI_95_) for total *Angiostrongylus* prevalence via the EpiTools calculator (https://epitools.ausvet.com.au/ciproportion).

A retrospective spatial analysis using SaTScan v10.1 (https://www.satscan.org) was conducted to detect spatial clusters of infected gastropods within the study area using the spatial scan statistic (Kulldorff and Nagarwalla 1995). The analysis was based on a binomial distribution, where 1 represented positive and 0 represented negative results, and used the Bernoulli model, under default parameters. The spatial cluster analysis was conducted at the grid level, with each gastropod treated as a separate observation and classified as either infected (case) or uninfected (control). Each gastropod's location was assigned to the centroid of the grid where it was collected, and these centroids were represented as Cartesian coordinates (*x*, *y*). Coordinates were entered into SaTScan using the Cartesian settings. Statistical significance was defined by a *p*‐value < 0.05.

### 2.5 Gastropod Digestion and L3 Isolation

Gastropods testing positive were individually digested to confirm the presence of *A. cantonensis* L3. Tissue samples were minced and incubated in a digestion solution containing 20 mL of water, 160 μL of HCl and 0.1 g of pepsin per gram of tissue. The mixture was heated at 45°C and agitated at 400 rpm for 30 min using a magnetic stirrer. The resulting solution was filtered through a 200 μm sieve and decanted in 50 mL centrifuge tubes. Larvae present in the precipitate were collected and identified based on morphological characters (Lv et al. 2009).

### 2.6 Sampling of Definitive Hosts

In addition to the initially detected positive rats, 19 cage rat traps baited with sausages were distributed in the study area. Due to land access restrictions, traps were only placed in some grids. Traps were monitored daily for 30 days. Captured rats were sexed and morphologically identified. Necropsies were conducted under biosafety guidelines at the University of the Balearic Islands, with examinations focused on detecting *A. cantonensis* in the lungs and heart.

### 2.7 Epidemiological Questionnaires

The two schools with children under 5 years old in the study area were invited to voluntarily participate in the survey. Both schools are situated in an urban setting, surrounded by vegetation. To maintain confidentiality, the specific names and locations of schools are not disclosed. To assess the potential risk of accidental *A. cantonensis* infection, a cross‐sectional study was conducted using an open‐ended epidemiological questionnaire for teachers, avoiding multiple‐choice formats to reduce response bias. Inclusion criteria required teachers who supervised children either outdoors or both indoors and outdoors. Face‐toto‐face interviews were conducted between January and February of 2024.

The epidemiological questionnaire consisted of three sections: (i) information on children's attitudes and practices, (ii) evaluation of specific risks and (iii) participants' knowledge of gastropod‐borne parasites, including *A. cantonensis* . The full epidemiological questionnaire is available in the Table S1.

## 3 Results

### 3.1 Prevalence of *A. cantonensis* and Risk Map Analysis

**FIGURE 1:**
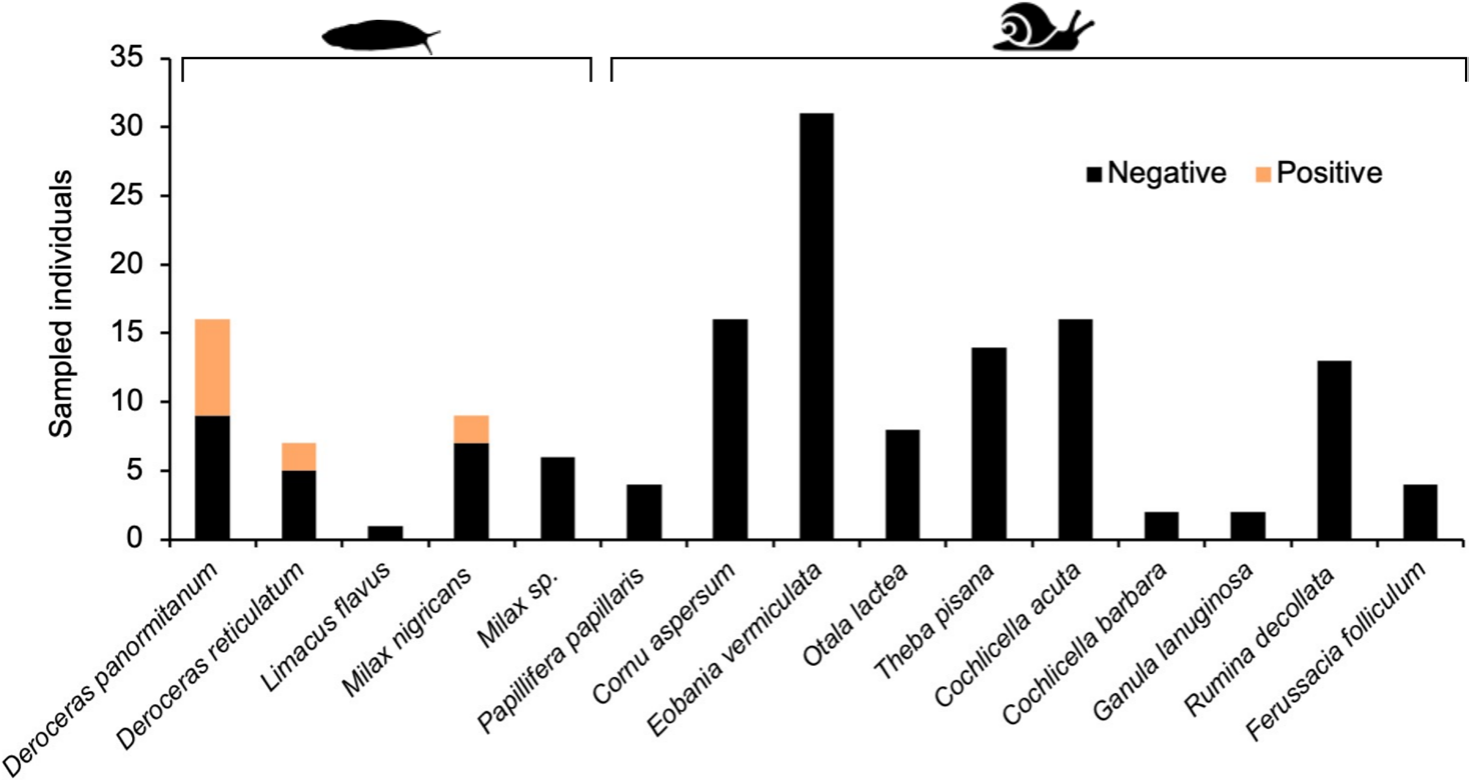
Stacked bar plot of infection status of gastropod species sampled in the 1 km radius study area according to AcanR3990 qPCR.

A total of 149 gastropods (110 snails and 39 slugs) representing 15 taxa were collected in 55 of the 68 complete grids from the study area (Figures 1 and S1). The overall *A. cantonensis* prevalence, as determined by AcanR3990 qPCR, was 7.38% (11/149, CI_95_ = 3.18%–11.58%). Positive gastropods belonged to three slug species: *Deroceras reticulatum* (*n* = 2), *D. panormitanum* (*n* = 7) and *Milax nigricans* (*n* = 2). Slugs only prevalence was 28.21% (11/39, CI_95_ = 14.08%–42.33%).

**FIGURE 2:**
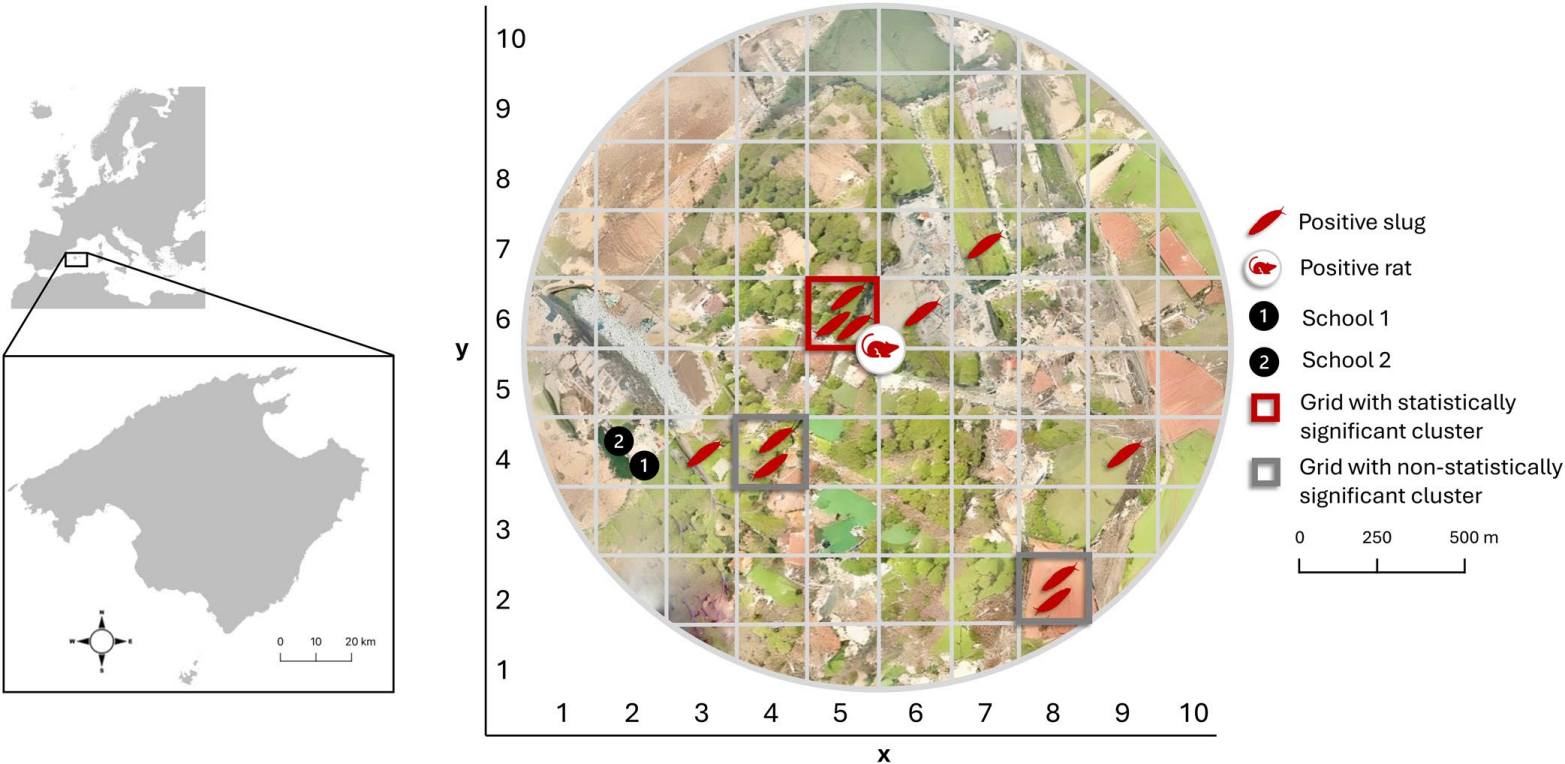
Study area of 1 km radius centred around the site where a positive rat was found during the *A. cantonensis* surveillance on the island of Mallorca. The area was divided into 200 × 200 m grids. Each slug icon represents a single positive slug. Specific locations and school names remain confidential.

Positive slugs were collected from seven grids within the study area, with the closest positive grid (*x* = 3, *y* = 4) situated ~200 m distant from the grid containing schools (Figure 2). Cluster analysis revealed three clusters in grids where more than one positive slug was found. However, only the cluster in grid *x* = 5, *y* = 6, which contained three positive slugs, was statistically significant (*p* = 0.023). In this grid, the relative risk of finding positive gastropods, as estimated by SaTScan, was 18.25 and is located surrounding the central point, where the positive rats were found (Figure 2).

In addition to the two positive rats used to construct the risk map, ten additional *Rattus* *rattus* specimens (6 males and 4 females) were sampled in the study area, but none tested positive for *A. cantonensis* .

### 3.2 Parasitic Load and Detection of L3 in Positive Slugs

**FIGURE 3:**
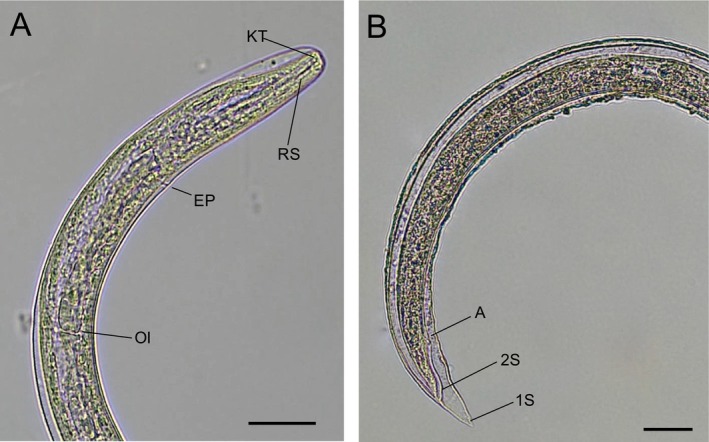
*Angiostrongylus cantonensis* L3 found in the AcanR3990 qPCR positive slugs after digestion. (A) Anterior part of the larvae with the knob‐like tips (KT), rod‐like structure (RS), excretory pore (EP) and oesophagus‐intestine junction (OI). (B) Posterior part of the larvae showing the anus (A), the two sheaths (1S, 2S) and the pointed tail. Scale bars: 20 μm.

After digestion of the positive slugs, larvae consistent with the morphology of *A. cantonensis* L3, as described by Lv et al. (2009) could be observed in at least one individual of each slug species (Figure 3). In some cases, larvae were damaged, likely due to freezing, which prevented the observation of all morphological characters.

**FIGURE 4:**
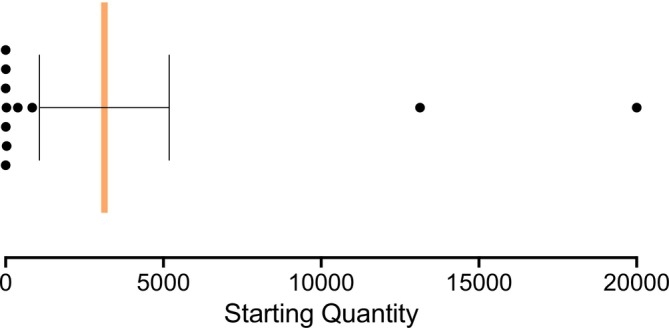
Larval burden estimation in AcanR3990 qPCR‐positive gastropod tissue snip DNA samples. The starting quantity of each sample according to five 10‐fold dilutions of known quantity of *A. cantonensis* L3s equivalent to 10, 1, 0.1, 0.01 and 0.001 × L3/s. The orange line indicates the mean, and the error bars show the standard error of the mean.

The larval load was quantified based on the starting quantity (SQ) obtained from the qPCR. The mean SQ for the 11 positive samples was 3131 × L3s (range = 0.708–20,000; Standard Error of the Mean = 2057; Figure 4). Most samples had an SQ below 100, while one *D. panormitanum* specimen (sample C149, Table S2) showed an extremely early qPCR amplification. In this case, an exact *C*_T_‐value and SQ could not be calculated and were recorded as 0 and a conservative ≥ 20,000 × L3 larvae, respectively. This slug was collected from the closest grid to the site where the positive rats were detected (*x* = 5, *y* = 6).

### 3.3 Epidemiological Questionnaires

Of the two schools in the study area, only School 2 agreed to participate, with all teachers (*n* = 4) completing the epidemiological questionnaires. Although School 1 allowed a meeting to discuss the study, they declined further involvement without providing additional explanations.

The responses to the epidemiological questionnaires from School 2 are provided in the Table S1. Key findings include that children spend between 2 and 3.5 h per day outdoors, rats have been observed in the vicinity of the centre and snails and slugs have been observed in the school playground areas. Several instances of children playing with snails and slugs were reported, and three out of four teachers confirmed witnessing children placing gastropods in their mouths (though to their knowledge, no children ingested them). Prior to this study, none of the teachers had any previous knowledge of rat lungworm or other gastropod‐borne diseases.

## 4 Discussion

This micro‐epidemiological study highlights the potential risk of neuroangiostrongyliasis in preschool children from an endemic site in Mallorca, a Mediterranean region where the parasite is well established. Although no autochthonous human cases have been reported in Spain—currently the only endemic country for the parasite in Europe—our pilot study confirms that *A. cantonensis* strongly circulates in slugs near the site where two positive rats were detected near children's outdoor spaces. The high parasitic load in some individuals and the presence of infective L3 confirm the intermediate host status of three slug species and underscore the imminent risk of disease transmission near preschool centres.

Out of the 15 gastropod species collected, only slug species *D. reticulatum* , *D. panormitanum* and *M. nigricans* tested positive for *A. cantonensis* (Figure 1) with *D. panormitanum* representing a new global host record. We confirm the role of these species as intermediate hosts for the parasite in Mediterranean Europe, as all three species were found to harbour L3. Interestingly, while the prevalence of *A. cantonensis* in slugs was 28.2%, none of the 110 snail specimens examined tested positive for the parasite. However, previous studies on the island have reported the circulation of the parasite in at least 11 gastropod species (Jaume‐Ramis et al. 2023).

The transmission risk of NA is influenced not only by the likelihood of ingesting gastropods or paratenic hosts harbouring L3, but also by the parasitic load present. The minimum infectious dose for *A. cantonensis* in humans remains uncertain. While a single larva—capable of growing up to 15 mm in the brain—could theoretically cause neurological disruption (Prociv et al. 2000), NA in humans is more likely the result of infection by several hundred or thousands of larvae, as deduced from experimental infections in other animals (Cowie 2013a). While most infected slugs in this study carried fewer than 40 larvae, two individuals harboured particularly high burdens, with 13,000 and ≥ 20,000 larvae, respectively. Notably, a slug carrying a high parasitic load (> 13,000 larvae) was found at ~200 m from a school. Several transmission pathways for *A. cantonensis* have been proposed (Cowie 2013b; Modrý et al. 2021), one of which involves the release of L3 through gastropod mucus. Although the number of larvae found in mucus is typically low, increased larval loads have been observed when gastropods experience stress (Rollins et al. 2023; Šipková et al. 2024).

Infected slugs were found in 7 of the 55 grids where gastropods were found; however, only one grid near the infected rats formed a statistically significant cluster (Figure 2). This grid also had the highest parasitic load (≥ 20,000 larvae), suggesting that infected rats may contribute to localised transmission risk. A potential limitation of the study is the relatively low and slightly variable number of gastropods sampled per grid, which could affect the statistical power to detect small spatial clusters. Nevertheless, sampling effort was standardised as much as possible across grids, and the Bernoulli model used in the spatial scan statistic appropriately accounts for case and control counts at each location. Given these limitations and the small study area, further studies are needed to confirm these findings.

In Sydney (Australia), a homogeneous *A. cantonensis* distribution with no significant clustering was observed using rat faecal samples as a proxy (Rivory et al. 2023). Despite their limited mobility, home ranges around 45 m^2^ have been defined for some slug species (Grimm and Paill 2001), potentially reaching outdoor areas frequented by children through time, particularly during wet seasons, when the risk of NA transmission is heightened (Delgado‐Serra et al. 2024; Hasan et al. 2025).

Autochthonous human cases are likely to emerge in Mallorca in the coming years, as it has occurred in other areas where raw gastropod consumption is uncommon. In endemic regions, outbreaks of rat lungworm disease have often occurred long after the parasite's initial detection. For instance, the first human cases of three children from Florida were reported two decades after the parasite was initially identified in captive wildlife (Chance et al. 2024). Similarly, in Sydney, the first report dates back to 1991 in dogs (Prociv et al. 2000), while human cases were not documented until 2001 (Senanayake et al. 2003).

Despite the limited scope of this pilot study, our findings confirm the convergence of several risk factors in an *A. cantonensis* endemic area of Mallorca, including (a) the presence of infected gastropods in proximity to schools, (b) high parasitic loads in some gastropods, reaching up to ≥ 20,000 larvae, (c) observations of children under five placing gastropods in their mouths and (d) the lack of awareness among caregivers responsible for child supervision. Given the severity of rat lungworm disease in children, including the risk of neurological sequelae (Evans‐Gilbert et al. 2014), and the current challenges for NA diagnosis (Hasan et al. 2025; McAuliffe et al. 2019), standardised protocols should be implemented in Europe. We recommend comprehensive surveillance to identify high‐risk areas in Spain where the parasite is known to circulate, enabling a more accurate assessment of transmission risk nationwide. Additionally, preventive strategies should be implemented in regions at high risk of infection, as early detection is crucial to improving patient prognosis.

## Author Contributions

S.J.‐R.: data curation, formal analysis, investigation, methodology, validation, visualization, writing – original draft, writing – review and editing. P.R.: formal analysis, investigation, methodology, validation, writing – review and editing. I.S.V.: investigation. J.Š.: methodology, validation, writing – review and editing. C.P.‐E.: conceptualization, funding acquisition, methodology, project administration, resources, supervision, validation, visualization, writing – review and editing.

## Ethics Statement

The epidemiological questionnaire developed in this study was reviewed and approved by the Ethics Committee of the University of the Balearic Islands. Gastropods were collected under the permission of the Species Protection Service of the Government of the Balearic Islands (CAP 16/2023). No specific permits were required for rat sampling as they are non‐protected species considered pests. Animal work was approved in accordance with the Spanish Government RD 53/2013 and the Biosecurity Committee of the University of the Balearic Islands.

## Conflicts of Interest

The authors declare no conflicts of interest.

## Supporting information


Data S1.

## Data Availability

The data that supports the findings of this study are available in the Data S1 of this article.
