# Supplementary material for: Neuroangiostrongyliasis Infection Risk Near Preschool Centres in Mallorca, Spain: A Pilot Micro‐Epidemiological Study
Source: Zoonoses Public Health. 2025 Jun 2;72(6):515–22. doi: 10.1111/zph.13228 (PMC12400015; doi:10.1111/zph.13228)

Supplementary material "**Neuroangiostrongyliasis infection risk near preschool centres in Mallorca, Spain: a pilot micro-epidemiological study**"

Supplementary Table S1. Epidemiological questionary developed in this study to assess the risk of infection with *Angiostrongylus cantonensis* in toddlers under 5-year-old from the schools located in the study area. Only school 2 accepted to participate in the study and all teachers (n=4) answered the questionary.

	i) Information on attitudes and practices of the children
**Teacher surveyed**	**1. How many times per day do the kids play in the outside during the scholar timetable?**
1	2 hours
2	2 hours
3	2 hours
4	3.5 hours
	ii) Evaluation of specific risks
**Teacher surveyed**	**2. Have you observed snails or slugs on the outdoor areas where children play?**
1	Yes
2	No
3	Yes
4	Yes
	**2.1. Please, specify where do you exactly saw the snails and slugs**
1	Close to the trees of the yard
2	-
3	In the garden, around the bushes
4	Around the trees
	**2.2. With which frequency have you observed the snails and slugs?**
1	At least once per week
2	-
3	Less than once per week
4	Every day
	**3. With which frequency have you observed the snails and slugs inside the building?**
1	Never
2	Never
3	Never
4	Never
	**4. Have you ever seen rats in the surroundings of the centre?**
1	Yes

2	No
3	No
4	Yes
	**5. With which frequency do you see children touching snails or slugs?**
1	At least once per month
2	Never in the last five years
3	At least once per month
4	Every day
	**6. Have you ever seen a child putting a snail or slug into their mouth?**
1	Yes
2	No
3	Yes
4	Yes
	**6.1. How long ago did you see that?**
1	More than two years ago
2	-
3	Two years ago
4	Last week
	**6.2. Where did it happen?**
1	In the yard of the school
2	-
3	In the garden
4	In the yard of the school
	**6.3. What happen after the incident?**
1	The child was scolded
2	-
3	Their hands and mouth were cleaned
4	The child was scolded
	iii) Participant's knowledge on gastropod-borne parasites
**Teacher surveyed**	**7. Do you know if snails and slugs can transmit any disease?**
1	No
2	Yes
3	No
4	No
	**7.1. Which kind of diseases?**
1	-
2	Do not know

3	-
4	-
	**8.Have you ever heard about the rat lungworm disease?**
1	No
2	No
3	No
4	No

Supplementary Table S2. Sample's information of the gastropods collected and AcanR3990 qPCR results, raw data.

Sample information		AcanR3990 qPCR				
**Sample ID**	**Species**	**Run**	**Ct-value**	**Cut-off Ct (Ct of 0.001 xL3)**	**qPCR result interpretation**	**Starting Quantity**
**C1**	*Ferussacia folliculum*	1	-	29,26	NEGATIVE	-
**C2**	*Ferussacia folliculum*	1	36,88289885	29,26	NEGATIVE	8,30E-06
**C3**	*Ferussacia folliculum*	1	-	29,26	NEGATIVE	-
**C4**	Deroceras panormitanum	1	-	29,26	NEGATIVE	-
**C5**	*Cornu aspersum*	1	-	29,26	NEGATIVE	-
**C6**	*Eobania vermiculata*	1	36,9429902	29,26	NEGATIVE	8,01E-06
**C7**	*Eobania vermiculata*	1	-	29,26	NEGATIVE	-
**C8**	*Otala lactea*	1	-	29,26	NEGATIVE	-
**C9**	*Cornu aspersum*	1	-	29,26	NEGATIVE	-
**C10**	*Cornu aspersum*	1	-	29,26	NEGATIVE	-
**C11**	*Cornu aspersum*	1	-	29,26	NEGATIVE	-
**C12**	*Eobania vermiculata*	1	38,23441379	29,26	NEGATIVE	3,68E-06
**C13**	*Milax sp.*	1	36,38989288	29,26	NEGATIVE	1,12E-05
**C14**	*Milax sp.*	1	-	29,26	NEGATIVE	-
**C15**	*Cochlicella acuta*	1	-	29,26	NEGATIVE	-
**C16**	*Ferussacia folliculum*	1	-	29,26	NEGATIVE	-
**C17**	*Cochlicella acuta*	1	37,36869477	29,26	NEGATIVE	6,20E-06
**C18**	Otala lactea	1	-	29,26	NEGATIVE	-
**C19**	*Rumina decollata*	1	-	29,26	NEGATIVE	-
**C20**	*Rumina decollata*	1	-	29,26	NEGATIVE	-
**C21**	*Deroceras reticulatum*	1	-	29,26	NEGATIVE	-
**C22**	*Theba pisana*	1	-	29,26	NEGATIVE	-
**C23**	*Theba pisana*	1	35,80873561	29,26	NEGATIVE	1,58E-05
**C24**	*Theba pisana*	1	-	29,26	NEGATIVE	-
**C25**	*Eobania vermiculata*	1	-	29,26	NEGATIVE	-
**C26**	*Cornu aspersum*	1	-	29,26	NEGATIVE	-
**C27**	*Theba pisana*	1	35,28873111	29,26	NEGATIVE	2,17E-05
**C28**	Limacus flavus	1	-	29,26	NEGATIVE	-
**C29**	*Theba pisana*	1	-	29,26	NEGATIVE	-
**C30**	*Eobania vermiculata*	1	34,5150713	29,26	NEGATIVE	3,45E-05
**C31**	*Cornu aspersum*	1	38,07281274	29,26	NEGATIVE	4,06E-06

Sample information		AcanR3990 qPCR				
Sample ID	Species	Run	Ct-value	Cut-off Ct (Ct of 0.001 xL3)	qPCR result interpretation	Starting Quantity
**C32**	*Cochlicella acuta*	1	-	29,26	NEGATIVE	-
**C33**	*Papillifera papillaris*	1	-	29,26	NEGATIVE	-
**C34**	*Papillifera papillaris*	1	-	29,26	NEGATIVE	-
**C35**	*Papillifera papillaris*	1	-	29,26	NEGATIVE	-
**C36**	*Rumina decollata*	1	-	29,26	NEGATIVE	-
**C37**	*Rumina decollata*	1	-	29,26	NEGATIVE	-
**C38**	*Eobania vermiculata*	1	-	29,26	NEGATIVE	-
**C39**	*Eobania vermiculata*	1	-	29,26	NEGATIVE	-
**C40**	*Theba pisana*	1	-	29,26	NEGATIVE	-
**C41**	*Theba pisana*	1	-	29,26	NEGATIVE	-
**C42**	*Theba pisana*	1	-	29,26	NEGATIVE	-
**C43**	*Deroceras reticulatum*	1	32,37484088	29,26	NEGATIVE	0,00012497
**C44**	*Deroceras reticulatum*	1	15,06939342	29,26	POSITIVE	4,147505498
**C45**	*Cochlicella barbara*	1	-	29,26	NEGATIVE	-
**C46**	*Cochlicella acuta*	1	-	29,26	NEGATIVE	-
**C47**	*Eobania vermiculata*	1	-	29,26	NEGATIVE	-
**C48**	*Papillifera papillaris*	1	34,94081458	29,26	NEGATIVE	2,67E-05
**C49**	*Cochlicella acuta*	1	33,89052633	29,26	NEGATIVE	5,02E-05
**C50**	*Cochlicella acuta*	1	-	29,26	NEGATIVE	-
**C51**	*Cochlicella acuta*	1	-	29,26	NEGATIVE	-
**C52**	*Milax sp.*	1	-	29,26	NEGATIVE	-
**C53**	*Rumina decollata*	1	37,86137003	29,26	NEGATIVE	4,61E-06
**C54**	*Cornu aspersum*	1	-	29,26	NEGATIVE	-
**C55**	*Cornu aspersum*	1	-	29,26	NEGATIVE	-
**C56**	*Cornu aspersum*	1	-	29,26	NEGATIVE	-
**C57**	Deroceras panormitanum	1	-	29,26	NEGATIVE	-
**C58**	*Cornu aspersum*	1	-	29,26	NEGATIVE	-
**C59**	*Eobania vermiculata*	1	-	29,26	NEGATIVE	-
**C60**	*Rumina decollata*	1	-	29,26	NEGATIVE	-
**C61**	*Eobania vermiculata*	1	37,61691715	29,26	NEGATIVE	5,34E-06
**C62**	*Milax nigricans*	1	-	29,26	NEGATIVE	-
**C63**	*Eobania vermiculata*	1	-	29,26	NEGATIVE	-
**C64**	*Milax nigricans*	1	-	29,26	NEGATIVE	-
**C65**	Deroceras panormitanum	1	-	29,26	NEGATIVE	-
**C66**	Deroceras panormitanum	1	-	29,26	NEGATIVE	-
**C67**	Deroceras panormitanum	1	11,64175093	29,26	POSITIVE	32,602086
**C68**	*Theba pisana*	1	-	29,26	NEGATIVE	-
**C69**	*Theba pisana*	1	-	29,26	NEGATIVE	-
**C70**	*Theba pisana*	1	-	29,26	NEGATIVE	-
**C71**	*Cochlicella acuta*	1	37,59890101	29,26	NEGATIVE	5,40E-06
**C72**	*Deroceras panormitanum*	1	-	29,26	NEGATIVE	-
**C73**	*Milax sp.*	1	-	29,26	NEGATIVE	-
**C74**	*Milax nigricans*	1	-	29,26	NEGATIVE	-
**C75**	*Milax nigricans*	1	-	29,26	NEGATIVE	-
**C76**	*Rumina decollata*	1	-	29,26	NEGATIVE	-
**C77**	*Rumina decollata*	1	-	29,26	NEGATIVE	-
**C78**	*Eobania vermiculata*	1	38,71194508	29,26	NEGATIVE	2,76E-06

Sample information		AcanR3990 qPCR				
Sample ID	Species	Run	Ct-value	Cut-off Ct (Ct of 0.001 xL3)	qPCR result interpretation	Starting Quantity
**C79**	*Milax sp.*	1	-	29,26	NEGATIVE	-
**C80**	Otala lactea	1	-	29,26	NEGATIVE	-
**C81**	*Otala lactea*	1	-	29,26	NEGATIVE	-
**C82**	*Cornu aspersum*	1	-	29,26	NEGATIVE	-
**C83**	*Theba pisana*	1	-	29,26	NEGATIVE	-
**C84**	*Cochlicella acuta*	1	-	29,26	NEGATIVE	-
**C85**	*Cochlicella acuta*	1	-	29,26	NEGATIVE	-
**C86**	*Cochlicella acuta*	1	35,17483279	29,26	NEGATIVE	2,32E-05
**C87**	*Eobania vermiculata*	1	37,3697167	29,26	NEGATIVE	6,19E-06
**C88**	*Cornu aspersum*	1	-	29,26	NEGATIVE	-
**C89**	*Rumina decollata*	1	-	29,26	NEGATIVE	-
**C90**	*Rumina decollata*	2	-	29,27	NEGATIVE	-
**C91**	*Eobania vermiculata*	2	35,18313288	29,27	NEGATIVE	1,79338E-05
**C92**	*Eobania vermiculata*	2	-	29,27	NEGATIVE	-
**C93**	*Eobania vermiculata*	2	-	29,27	NEGATIVE	-
**C94**	*Eobania vermiculata*	2	-	29,27	NEGATIVE	-
**C95**	*Cornu aspersum*	2	-	29,27	NEGATIVE	-
**C96**	*Cornu aspersum*	2	-	29,27	NEGATIVE	-
**C97**	*Cornu aspersum*	2	-	29,27	NEGATIVE	-
**C98**	*Eobania vermiculata*	2	-	29,27	NEGATIVE	-
**C99**	*Eobania vermiculata*	2	36,3789852	29,27	NEGATIVE	8,73834E-06
**C100**	*Eobania vermiculata*	2	-	29,27	NEGATIVE	-
**C101**	*Deroceras reticulatum*	2	-	29,27	NEGATIVE	-
**C102**	*Eobania vermiculata*	2	-	29,27	NEGATIVE	-
**C103**	*Eobania vermiculata*	2	-	29,27	NEGATIVE	-
**C104**	*Eobania vermiculata*	2	30,62371713	29,27	NEGATIVE	0,000278076
**C105**	*Deroceras panormitanum*	2	37,31255374	29,27	NEGATIVE	4,98504E-06
**C106**	*Deroceras panormitanum*	2	1,232567607	29,27	POSITIVE	13133,18784
**C107**	*Ganula lanuginosa*	2	-	29,27	NEGATIVE	-
**C108**	*Deroceras panormitanum*	2	14,11520652	29,27	POSITIVE	5,683757423
**C109**	*Deroceras panormitanum*	2	14,98199751	29,27	POSITIVE	3,375290715
**C110**	*Deroceras panormitanum*	2	-	29,27	NEGATIVE	-
**C111**	*Deroceras panormitanum*	2	-	29,27	NEGATIVE	-
**C112**	*Milax sp.*	2	-	29,27	NEGATIVE	-
**C113**	*Rumina decollata*	2	-	29,27	NEGATIVE	-
**C114**	Eobania vermiculata	2	-	29,27	NEGATIVE	-
**C115**	Otala lactea	2	-	29,27	NEGATIVE	-
**C116**	*Deroceras reticulatum*	2	38,3395252	29,27	NEGATIVE	2,68856E-06
**C117**	*Deroceras reticulatum*	2	-	29,27	NEGATIVE	-
**C118**	Otala lactea	2	-	29,27	NEGATIVE	-
**C119**	Otala lactea	2	-	29,27	NEGATIVE	-
**C120**	Eobania vermiculata	2	-	29,27	NEGATIVE	-
**C121**	Cochlicella barbara	2	-	29,27	NEGATIVE	-
**C122**	*Cochlicella acuta*	2	-	29,27	NEGATIVE	-
**C123**	*Milax sp.*	2	-	29,27	NEGATIVE	-
**C124**	*Milax nigricans*	2	7,098580777	29,27	POSITIVE	386,1168744
**C125**	*Milax nigricans*	2	-	29,27	NEGATIVE	-

Sample information		AcanR3990 qPCR				
**Sample ID**	**Species**	**Run**	**Ct-value**	**Cut-off Ct (Ct of 0.001 xL3)**	**qPCR result interpretation**	**Starting Quantity**
**C126**	*Milax nigricans*	2	-	29,27	NEGATIVE	-
**C127**	*Cochlicella acuta*	2	37,0299736	29,27	NEGATIVE	5,90815E-06
**C128**	*Cochlicella acuta*	2	-	29,27	NEGATIVE	-
**C129**	*Theba pisana*	2	-	29,27	NEGATIVE	-
**C130**	*Deroceras panormitanum*	2	-	29,27	NEGATIVE	-
**C131**	*Cornu aspersum*	2	-	29,27	NEGATIVE	-
**C132**	*Ganula lanuginosa*	2	-	29,27	NEGATIVE	-
**C133**	Otala lactea	2	-	29,27	NEGATIVE	-
**C134**	Eobania vermiculata	2	-	29,27	NEGATIVE	-
**C135**	Eobania vermiculata	2	-	29,27	NEGATIVE	-
**C136**	*Cochlicella acuta*	2	37,82232109	29,27	NEGATIVE	3,66915E-06
**C137**	*Cochlicella acuta*	2	-	29,27	NEGATIVE	-
**C138**	*Eobania vermiculata*	2	-	29,27	NEGATIVE	-
**C139**	*Theba pisana*	2	-	29,27	NEGATIVE	-
**C140**	*Eobania vermiculata*	2	-	29,27	NEGATIVE	-
**C141**	*Eobania vermiculata*	2	-	29,27	NEGATIVE	-
**C142**	*Eobania vermiculata*	2	-	29,27	NEGATIVE	-
**C143**	*Rumina decollata*	2	-	29,27	NEGATIVE	-
**C144**	*Rumina decollata*	2	-	29,27	NEGATIVE	-
**C145**	*Milax nigricans*	2	5,798374177	29,27	POSITIVE	843,7437273
**C146**	*Deroceras reticulatum*	2	13,92677802	29,27	POSITIVE	6,365541465
**C147**	*Deroceras panormitanum*	2	11,3674277	29,27	POSITIVE	29,65494589
**C148**	*Deroceras panormitanum*	2	17,57810048	29,27	POSITIVE	0,708684707
**C149**	*Deroceras panormitanum*	2	0	29,27	POSITIVE	20000

Supplementary Figure S1. Number of gastropods sampled in each grid of the study area.

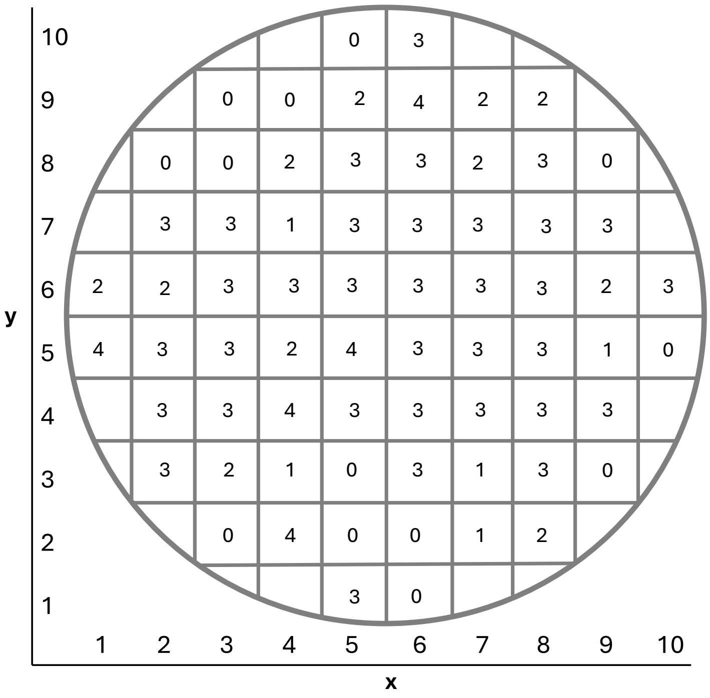

Supplement: Supplementary file 1 — Data S1. [file ZPH-72-515-s001.pdf]
